# Why Do They Leave? Challenges to Retention of Surgical Clinical Officers in District Hospitals in Malawi

**DOI:** 10.34172/ijhpm.2020.142

**Published:** 2020-08-05

**Authors:** Jakub Gajewski, Marisa Wallace, Chiara Pittalis, Gerald Mwapasa, Eric Borgstein, Leon Bijlmakers, Ruairi Brugha

**Affiliations:** ^1^Institute of Global Surgery, Royal College of Surgeons in Ireland, Dublin 2, Ireland.; ^2^Faculty of Health, Medicine and Life Science, Maastricht University, Maastricht, The Netherlands.; ^3^Division of Population Health Sciences, Royal College of Surgeons in Ireland, Dublin 2, Ireland.; ^4^Department of Surgery, College of Medicine Malawi, Blantyre, Malawi.; ^5^Radboud University Medical Centre, Nijmegen, The Netherlands.

**Keywords:** Non-physician Clinicians, Task-Sharing, Global Surgery, Malawi

## Abstract

**Background:** Low- and middle-income countries (LMICs) are the worst affected by a lack of safe and affordable access to safe surgery. The significant unmet surgical need can be in part attributed to surgical workforce shortages that disproportionately affect rural areas of these countries. To combat this, Malawi has introduced a cadre of non-physician clinicians (NPCs) called clinical officers (COs), trained to the level of a Bachelor of Science (BSc) in Surgery. This study explored the barriers and enablers to their retention in rural district hospitals (DHs), as perceived by the first cohort of COs trained to BSc in Surgery level in Malawi.

**Methods:** A longitudinal qualitative research approach was used based on interviews with 16 COs, practicing at DHs, during their BSc training (2015); and again with 15 of them after their graduation (2019). Data from both time points were analysed and compared using a top-down thematic analysis approach.

**Results:** Of the 16 COs interviewed in 2015, 11 intended to take up a post at a DH following graduation; however, only 6 subsequently did so. The major barriers to remaining in a DH post as perceived by these COs were lack of promotion, a more attractive salary elsewhere; and unclear, stagnant career progression within surgery. For those who remained working in DH posts, the main enablers are a willingness to accept a low salary, to generate greater opportunities to engage in additional earning opportunities; the hope of promotional opportunities within the government system; and greater responsibility and recognition of their surgical knowledge and skills as a BSc-holder at the district level.

**Conclusion:** The sustainability of surgically trained NPCs in Malawi is not assured and further work is required to develop and implement successful retention strategies, which will require a multi-sector approach. This paper provides insights into barriers and enablers to retention of this newly-introduced cadre and has important lessons for policy-makers in Malawi and other countries employing NPCs to deliver essential surgery.

## Background

**Key Messages**

** Implications for policy makers**

- The rural retention of non-physician surgical staff needs to be prioritised within health policy in low-income countries such as Malawi.
- Lack of promotion, low salary and recognition, unclear career paths and poor working conditions fuel the attrition of non-physician surgical staff from the public system in low-income countries and need to be addressed through a multi-sector approach in order to implement successful retention strategies.
- A Bachelor of Science (BSc) in surgery has made these clinical officers (COs) more employable to other institutions and at higher levels; hence policy-makers need to develop surgical non-physician clinician (NPC) career pathways in order to increase retention at the district level, ie, ‘carrots,’ rather than using traditional methods to curb internal migration to urban areas (‘sticks’).
- Policy-makers should be aware of the need to develop and implement an improved model for collaboration between non-governmental organisations (NGOs) and local health systems in low- and middle-income countries (LMICs). In such cases where it is required to hire local health-workers, wages should be in line with those offered by local institutions so as not to distort the labour market.

** Implications for the public**

Programmes developing specialist training for non-physician clinicians (NPCs) upgrading them to BSc (Bachelor of Science) level, need to include strategies to retain them in the facilities in which recent evidence has demonstrated that essential surgery for surgical problems, that are commonly encountered in rural African countries, can be delivered safely and effectively by surgically trained NPCs. However, strategies are needed, in countries such as Malawi, to retain and create career paths for this essential type of surgically trained health worker.

Common challenges affecting surgical productivity in low- and middle-income countries (LMICs) are staff shortages, availability of essential infrastructure and supplies, and low priority of surgical care within health policies.^1^ The lack of sufficient numbers of trained surgical providers is among the most significant barriers,^2-5^ particularly in sub-Saharan Africa (SSA).^6^ Given the chronic shortage of surgeons in SSA, non-physician clinicians (NPCs) are trained to perform essential surgical tasks.^7^ NPCs are health-workers who have not been trained to the level of a medical doctor but take on some of their diagnostic and clinical functions.^8^ They are hailed as an alternative due to low training costs, reduced training duration and better retention rates in rural placements compared to medical doctors.^9^ The shifting of tasks to lower cadres, such as NPCs, when safe and reasonable is endorsed and recommended by the World Health Organization (WHO).^10,11^

Surgical care provision in Malawi relies on NPCs, as the density of specialist surgeons sits at 0.23 per 100 000 people, the lowest in the world.^12^ NPCs are essential in district hospitals (DHs) in rural areas, where 83% of Malawians reside,^13^ as specialist surgeons are exclusively employed at central hospitals. With an ever-expanding population (increase of over 31% over the last 10 years^14^) and increasing rates of non-communicable diseases,^15^ compounded by its economic instability, the strain on the Malawian health system to address the surgical disease burden remains profound. A survey by Varela et alidentified that approximately one-third of the Malawian population is living with a surgical condition that requires consultation,^16^ reinforcing the need for urgent scale-up of surgical services.^7^ In Malawi NPCs are known as clinical officers (COs) and possess a Diploma in General Medicine. In recent years, programmes have been developed to upgrade NPC-training to Bachelor of Science (BSc) level to develop advanced skills in several disciplines.^7,9,17,18^ One such programme was the Clinical Officer Surgical Training (COST)-Africa project. In 2011-2016 COST-Africa developed and implemented a BSc course in general surgery at the College of Medicine, Malawi. The first cohort of students included 17 COs, who were trained in common emergency and elective procedures ‘on-the-job’ through a placement in 8 DHs. The objective of COST-Africa was to launch a training model producing surgical providers equipped with skills and knowledge adequate to the needs of rural dwellers. An evaluation of the programme by Gajewski et al^7^ demonstrated its effectiveness in improving surgical productivity at DHs in Malawi.

As seen in other cadres of health-workers worldwide,^19,20^ training these COs is merely the first step towards addressing surgical staff shortages. Another major issue is migration, whether it be external migration from low to higher-income countries or internal migration from rural to urban areas.^21^ This “brain drain”^22^ is particularly rife in SSA^23^ and affects not only highly-trained healthcare professionals, but also mid-level cadres such as NPCs. There are growing numbers of studies describing factors influencing the retention of health-workers in rural areas and proposing potential interventions, but without analysing the effects of these interventions.^24^ This study contributes to building a more robust evidence in this field, by assessing the effects of the COST-Africa intervention on the retention of surgically-trained NPCs in rural areas. Although set in Malawi, this study aims to provide important policy lessons for all countries who employ (or intend to employ) specialised NPCs to address the gap in their surgical workforce.

## Methods

###  Study Design

This study is reported using the COREQ checklist for qualitative studies.^25^ A longitudinal qualitative research^26^ method was used, based on narrative semi-structured interviews with the first cohort of COST-Africa COs (CA-COs) conducted at 2 time points: (1) November-December 2015, while they were still undergoing the COST-Africa BSc programme; and (2) June-July 2019, three years after graduation. The initial interviews were conducted to investigate participants’ experience of the training programme and their career plans following graduation, as well as to document wider issues affecting surgery at DHs (as reported elsewhere^27^). The second set of interviews explored factors affecting the retention of CA-COs in public DHs after obtaining a BSc in Surgery. The 2015 interviews were conducted using a topic guide developed based on a literature review and the researchers’ experience of working with NPCs in Malawi. The second guide was similarly composed, additionally informed by the findings of the prior interviews. This longitudinal qualitative design offers an opportunity to engage with the complex and multifactorial nature of the retention of surgically trained NPCs at the district level in Malawi. By following individual trajectories, the factors influencing CA-COs’ decisions were identified as well as changes in how these self-declared factors acted as barriers or enablers to working at DHs.

###  Participants’ Recruitment/Consent

 All 17 CA-COs who took part and completed the COST-Africa training were contacted via email before the interviews. Of these, 16 were able to participate in the first interviews and 15 in the second round. No incentive was provided. The individuals who did not take part in the study were unavailable at the time the interviews were conducted.

###  Data Collection 

 Interviews lasted on average 50 (2015) and 30 minutes (2019). Participants were given information about the purpose of the interview beforehand. They were informed about the aim of the study and their consent was obtained. All interviews took place face-to-face at a location convenient to the participant, with 2 project researchers present (2015: JG, GM; 2019: JG, MW). JG (male) is a senior researcher with extensive training and experience in qualitative methods, GM (male) and MW (female) were junior qualitative researchers at the time of the study.

###  Data Analysis

A narrative methodological approach was followed^28^ to explore changes in career trajectories of participants. The recorded interviews were transcribed using MS Word. Data analysis was carried out using a ‘top down’ thematic approach. The coding matrix for the 2015 data was informed by personal experience of the research team (JG and GM), interacting with CA-COs during the COST-Africa training and evaluation, and by a thorough literature review. This coding matrix was modified after analysis of the first batch of data. Subsequently all 2015 interviews were coded by one researcher (MW) according to the final matrix and an initial set of themes were drafted. Coding was then verified and discussed with a senior researcher (JG) to agree upon the themes. The 2019 interviews were analysed in a similar manner, informed by the completed analysis of interviews conducted in 2015. Two additional steps were added: data from 2019 were compared with the 2015 to further refine the coding matrix and at the last stage of the analysis all themes were discussed with a third researcher (CP) to verify and agree the final structure.

## Results

###  Career Intentions Before Graduation (2015)

 Information regarding past and present employment, as well as employment intentions at the time of COST-Africa are reported in Table. Before starting the BSc programme 11 CA-COs were working in DHs, 2 were working in central hospitals, 3 in mission hospitals and 1 in an NGO. During the COST-Africa BSc programme all participants were stationed at DHs for a period of 24 months. This was a key feature of the training as the scope was to prepare COs to work in district surgical teams. Of the 16 CA-COs interviewed towards the end of the placement in 2015, 11 expressed intent to take up or continue in a post at a DH. Among these, there was a sense of duty to help patients in remote areas.

**Table:** Employment Status of Study Participants Before, at the Time and After the COST-Africa Programme and Employment Expectations Upon Completion of It

	**Employment Before COST-Africa**	**Employment Intentions During COST-Africa (2015)**	**Employment After Graduation From COST-Africa (2019)**
CO_1	CH	CH	CH
CO_2	MDH	GDH	NGO
CO_3	GDH	GDH	GDH
CO_4	GDH	GDH	GDH
CO_5	GDH	GDH	GDH
CO_6	MDH	MDH	MDH
CO_7	GDH	Not interviewed	NGO
CO_8	GDH	GDH	NGO
CO_9	CH	CH	GDH
CO_10	MDH	MDH	Not interviewed
CO_11	NGO	CH	NGO
CO_12	GDH	GDH	NGO
CO_13	GDH	GDH	GDH
CO_14	GDH	GDH	MoH HQ
CO_15	GDH	GDH	Academia^a^
CO_16	GDH	GDH	Not interviewed
CO_17	GDH	GDH	GDH

Abbreviations: COST, Clinical Officer Surgical Training; CH, central hospital; MDH, mission district hospital. GDH, government district hospital; NGO, non-governmental organisation; MoH HQ: Ministry of Health Head Quarters. ^a^Academia: lecturing at a university.

“*…when I went to DH X, I found out that most of the surgical cases were kind of mistreated. I feel like if I can go back to the district I will be able to…change things” *(CO6, 2015).

 Despite this, only 6 (35%) had chosen to do so and remained surgically active by the time of the second round of interviews in 2019. Out of the 6 who were not employed at DHs before COST-Africa, only 1 took up a position at a DH following graduation.

###  Factors Affecting Retention of CA-COs at District Level

Figure illustrates the themes around factors affecting retention and attrition of CA-COs at DHs as emerging from the interviews in this study. Two groups of inter-linked factors are at play: (*i*) organisational factors (at level of individual health facilities); (*ii*) systemic factors ie, strategies (or lack of) employed by the government, hospital management or other institutions. Both sets of factors can act as *push* or *pull* forces.

**Figure:**
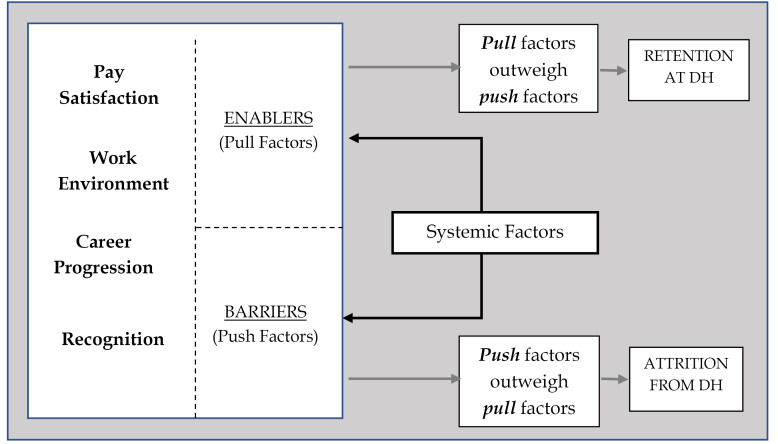
Emerging themes. Abbreviation: DH, district hospital.

####  Pay Satisfaction

 Remuneration emerged as one of the most dominant themes affecting CA-COs work choices. During their BSc in surgery training (2015), most CA-COs assumed that upon graduation they would be automatically promoted, with a salary increase reflecting their higher qualifications compared to the diploma level of other general COs.

“*They promised us as soon as we finished our school they are going to promote us. That is our expectation because that is what the minister for health promised us” *(CO9, 2015).

 When this was only granted to 2 CA-COs (as reported in the 2019 interviews), many respondents experienced frustration. Their workload after graduation remained the same, or increased in some instances, while the remuneration package did not change.

“*The salary is not good enough. Our salary is not even enough to manage to get food to last through the month*...*So that’s why most of the people we have found have left the district..*.” (CO14, 2019).

“*…Where do we get if we do all the surgeries and then we still get the same old salary, even though we went to school...the pay-checks remained the same while the responsibilities were expanding…” *(CO8, 2019).

 A better salary in the NGO sector was cited as the primary reason for taking up alternative employment, often no longer using their surgical skills. COs working outside the DH were more commonly engaged in the management of teams or administrative duties, with only a few, at best, undertaking minor procedures such as circumcision.

“*Because when you are in diploma level you are at 180-190k [Malawian Kwacha ~$250/month]. While on degree level is 290 something to 300k [Malawian Kwacha ~$400/month]. So, they are quite different from what you would earn at NGOs, compared to the government hospitals”* (CO3, 2019).

Respondents also reported that surgery as a field did not hold many opportunities for additional income, which are commonly sought by health-workers in LMICs to make up for low salaries. Such opportunities often come in the form of training, workshops or educational seminars with financial compensation provided by the organisers or government.^29^ While these were common in areas such as malaria, tuberculosis, maternal and child health, and HIV, there were none specific to surgery at the time of COST-Africa. In addition, some respondents felt that due to their workload, they were less likely to be offered or given permission to avail of these opportunities even if they were to be made available.

“*…if you are doing a BSc in paeds there is a lot of trainings. You go to so many trainings. As surgeons, you spend your time in theatre. So, surgery you spend working while the others make money by doing some trainings … So, I think a lot of people dislike surgery because of that. That’s my drawback”* (CO3, 2019).

“*…some people would maybe rather go and do paediatrics or go do obs and gynae because they say maybe it’s more marketable than surgery”* (CO13, 2019).

 Despite these constraints, many respondents felt that the surgical training and qualification offered them more chances to find additional earning opportunities working at the district level. At central hospitals some respondents felt there were more people to compete with and in the private sector, such as NGOs, respondents described less flexibility to do so. Study participants felt that they had more independence in the public sector. This was seen as a positive factor by the 6 CA-COs who stayed at the district level.

“*Basically, at the district hospital despite that you are tight with surgery, sometimes because you know our earnings are small, you can do some other activities…You know for certain at the central level it is dependent on the approval from the consultant”* (CO5, 2019).

“*Working for the government, yes, we sweat, but we’re flexible. We can do other things. But when you’re working in the private sector, you can’t do anything” *(CO13, 2019).

####  Work Environment

 Respondents to the 2019 interviews felt inadequately supported in theatre due to either low motivation or the lack of skills of other team members. This was another source of frustration working in DHs, with some respondents feeling unable to work to their potential. Some also noted that this affected workload. The higher qualifications of the CA-COs who completed the BSc programme meant that other colleagues (general COs and anaesthetists) dismissed many surgical cases, regardless of complexity, leading to unnecessarily higher workload for the CA-COs.

“*But in district hospitals we can also say there is some workload because there are few technicians who are doing these surgical cases, a lot of people leave these surgical cases for us, basically because we’ve done the BSC in General Surgery. So even minors, they just leave it to me”* (CO3, 2019).

 Another issue was the lack of resources and/or infrastructure to meet demand for surgical services at the DH. This often caused postponement or cancellation of operations, leading to a backlog of patients and increased workload for the CA-COs. Respondents who had left the public sector, expressed the benefits of no longer having to face these issues.

“*And also, if you look at the resources, if you request for something *[at the NGO]* you are sure that you are going to get it. Unlike in the government, where you are going to have to improvise. Sometimes even if you improvise you end up failing what you wanted to do” *(CO7, 2019).

 Despite the reports of substantial workload at DHs, there appeared to be a near consensus among respondents that this was even greater at central hospitals. For this reason, many felt that they would still prefer to work at the district level.

“*…at central hospital there’s even much pressure of work. Even if when you are given a chance there’s no time to rest. Whereas in a district hospital, maybe they might sleep the whole night, even if they are on call. Or maybe they do one or two operations and they are done”* (CO1, 2019).

####  Career Progression

 A major cause of discontent among the CA-COs was the lack of career advancement opportunities available to them within the surgical field as a non-physician, causing the attrition of many from DHs and indeed surgery itself. When interviewed in 2015, respondents hoped that by completing the BSc in Surgery, a path would be developed for them to continue education to masters level with a view to advancing their surgical career. There were also deliberations about the possibility of a bridging programme being put in place allowing graduate entry to the Bachelor of Medicine or/and Bachelor of Surgery (MBBS), reducing the usual time of 5 years.

“*…I want to be a doctor.” “ Yeah this is like a stepping-stone towards that. I know in the future after I do this, doors will open and everything. Maybe MBBS or something. But I want to end up being a doctor…maybe in the future there might be a bridging program. If you do a BSc in surgery…MBBS program after surgery for you” *(CO16, 2015).

 However, when this did not materialise, by 2019 respondents felt let down. Many described feeling ‘forced’ to divert into other careers, despite surgery being where their preference laid. Many CA-COs intended to move or had already moved into fields such as public health or epidemiology, where options to pursue a master’s degree were available to BSc-holders.

“*I’ve even applied for the MPH…It’s just that I’ve been stagnant for so long…If we had the opportunity to do masters in the same field as surgery…that would have been much better for us” *(CO17, 2019).

 Among those who remained in employment at the district level, there was still hope that opportunities would come with time, believing that their length of service in the public sector would eventually be rewarded with a promotion or chances to take time off to study.

“*Basically, myself I’ve already served for the Ministry for a good number of years…I still wish, and I still have that hope that possibly one day the Ministry might recognise us *[with a promotion]*”* (CO4, 2019).

“*You know, one thing that keeps me around is maybe one day an opportunity comes for maybe school. You know, when you’re in the government it’s easier to do school than doing it when you’re in the private sector. You know in the private sector, time is more important than in the government. In the government we can go back to school for some time and go back”* (CO13, 2019).

####  Recognition

 On a positive note, most respondents seemed to appreciate the greater responsibilities given to them by the DH management after completing their BSc course. They felt they had a greater decision-making role within the surgical department and were the “go-to” member of staff for all surgical-related matters. For some of them this was a reason for preferring to work at the district rather than the central level. Respondents explained that at a central hospital they would not have the same level of recognition or responsibility because specialists and surgical trainees usually had priority over access to the operating theatre and opportunities for practicing their surgical skills.

“*You know actually when I was in *[the DH]* I was the big man...Because each and every surgery that comes around I was the final decision-maker...So that is what made me to think that at least I was recognised ”* (CO15, 2019).

“*I think the district hospital offers us a greater opportunity to practice compared to the central hospital. In central hospitals there are more surgeons and to be given the chance to practice at the central hospital the chances are slim compared to the district” *(CO4, 2019).

 However, the recognition of the CA-COs new qualifications, skills and responsibilities was often limited, as demonstrated by a general lack of job promotions. Discontent among many respondents was also due to the lack of recognition in the form of an official professional title to distinguish them from the other general COs at diploma level. These factors had a negative impact on their motivation to remain working at the DH and in surgery.

“*We are called clinical officers. We went for BSc. We are still called clinical officers. So, we are appealing that we should at least be called the name that we suggested, associate specialist” *(CO8, 2019).

“*I still want to at least be practicing and be more like in my, in the line that I can upgrade myself easily than what I’m doing now. Because now the bachelor’s is not working at all, because I was employed and I have the Diploma, so it’s like I wasted years being in school obtaining a paper which I’m not using”* (CO2, 2019).

 On the contrary, the BSc in surgery was well regarded by other institutions, such as NGOs. Those respondents who took up employment in NGOs felt that the BSc gave them an advantage in securing their position. One respondent stated that holding a BSc was an essential requirement for her role.

“*…myself having a degree, I was at an advantage. And that’s why I’m holding that post. I have my subordinates, I’m a team leader of 11 people …Why? Because we’re having the BSc”* (CO8, 2019).

## Discussion

As shown by the evaluation of the COST-Africa intervention^7^ and in other studies,^30^ NPCs such as CA-COs are essential in scaling up surgery in many SSA countries. COST-Africa was effective in achieving what it set out to do, namely by training a cadre of health-workers able to improve surgical productivity in DHs and willing to practice in rural areas. Most respondents appreciated their new skills and responsibilities, and had a genuine interest in a surgical career within the public DH, as shown by their hopes and enthusiasm at the time of their studies. Those who continued working at the DH after the completion of the BSc enjoyed their new roles as reference point for the local surgical teams and the greater responsibility within the surgical department, despite the increased workload. Existing challenges included the lack of official recognition by the health system in the form of either promotion (and associated pay scale advancement) or change in job title to differentiate COs BSc holders from the other general COs with diploma qualifications. We hypothesise that the changeover in the Malawian government that took place during COST-Africa may have played a role in this lack of follow up.

According to equity theory, employees seek to achieve a balance between what they put into their jobs in terms of effort, knowledge and skills (inputs), and what they get as an outcome through compensation or recognition (outputs).^31,32^ There is also a documented concern that quality of care may suffer when health-workers are not properly supported and motivated.^33-37^ Findings from this study suggest that perceived imbalances and pay dissatisfaction among respondents when working in public DHs resulted in reduced motivation. In order to restore equity the value given to additional earning opportunities, such as workshops, increased and some CA-COs were pushed to seek work in other, more profitable sectors, such as NGOs, triggering intention of other respondents to follow suit.

In Malawi, and other countries in the SSA region, allowances from sponsored training programmes are for many a substantial part of a monthly take-home salary, and can influence their overall pay satisfaction.^38^ Findings from this study suggest that surgery as a profession is not regarded as highly as other medical specialties, specifically in regards to concerns over lack of opportunity to access other sources of income specific to the surgical discipline, namely ‘training’ and ‘workshops.’ Respondents suggested that other specialities such as paediatrics offer greater opportunities to top-up a government salary through participation in such aforementioned activities and generally perceived as more ‘profitable.’^39^ This reflects the trend in Malawi and other LMICs whereby surgery receives relatively little consideration within health systems and health policy. Resources invested and subsequent training and additional earning opportunities available for staff in surgery are therefore considerably less compared to other areas such as infectious disease^3^; and compared with donor, externally funded initiatives.^40^

As one of the poorest countries in the world, Malawi relies heavily on international aid^41,42^ often channelled through international NGOs.^43,44^ This can account for the surge in NGOs operating in the country and subsequent spikes in movement of health-workers from the public health-system to these institutions.^45^ Despite intentions of supporting the local health system, these trends can inadvertently undermine the health system.^43,46^ The evidence from our study supports the call by other researchers to develop and implement an improved model for collaboration between NGOs and local health systems in LMICs.^47^ In such cases, where it is required to hire local health-workers, wages should be in line with those offered by local institutions so as not to distort the labour market. NGOs should also consider investing in the education and training of professionals which they are removing from public service, to help increase the number of health staff in the country overall.^47^

The barrier of perceived stagnancy and lack of clarity around the NPC career path within surgery is an issue reported in other studies based in Malawi on a range of health-workers, and in other countries.^36,38,48,49^ According to the Lancet Commission on Global Surgery, defining and developing career pathways for mid-level cadres is essential for successful retention.^10^ However, the lack of career structure for NPCs has resulted in the widespread perception that, at least in Malawi, they are trained to a level at which they are useful, and then abandoned.^36^ The introduction of a BSc has not been enough to curb attrition of COs out of the public sector into other professions with better career progression opportunities, with many CA-COs in the 2019 interviews questioning the longer-term career prospects for them within the surgical field. McAuliffe posited problems with CO training and career structures in Malawi that may have been overlooked and that addressing these factors are essential to retention of COs^34^; and our study supports this conclusion. A major take-away point from this study for policy-makers is the fact that obtaining a BSc makes these COs more employable to other institutions; thus focus should be shifted to developing the surgical NPCs career pathway in order to increase retention at the district level.

The findings in this study highlight the underlying issue in Malawi of poor or lack of planning, management and support to the health workforce – problems commonly seen in LMICs. Reviewing the distribution of surgical NPCs in Malawi, accompanied by reviewing surgical-needs assessments, like that of Varela et al,^16^ would allow strategic planning by the Ministry of Health (MoH) in the allocation of staff and allow for better planning of student intake and promotional opportunities. It also must be noted that the developments in CO deployment in the surgical workforce should occur in parallel with strategic rethinking about the role of physicians and with critical innovations in specialist surgeon recruitment and in-service training.^30^ Successful implementation of a comprehensive human resources plan needs the collaboration and commitment of a multi-sectoral group,^45^ most importantly in this case between the Malawian Government and NGO sector, with further studies on monitoring and ensuring appropriate codes of conduct among NGOs required.

###  Study Limitations

 The primary limitation is the limited generalisation of these results to other countries and healthcare settings as this study is based on participants from Malawi. They have been exposed to challenges that may be specific to the local context and thus may not apply to similar surgical NPCs in other countries. For example, the rates of pay and promotional opportunities may vary from country to country. However, for the majority it is believed that the themes identified within this study that act as barriers to retention of non-physician surgical staff are also transferable to other LMICs, particularly in SSA due to the homogeneity of healthcare problems in the region. Secondly, this study did not seek out the views of other stakeholders implicated in the discussion of retention of these COs, such as the MoH and NGOs. This was consistent with the aim of the study, which was to gain insight and understanding of the COs perceptions of barriers and enablers to retention at the DH. The discourse surrounding the impact of the influx of NGOs on public-health systems could be strengthened by way of further studies on this issue, including NGO and government perspectives. A third limitation can arise due to the self-reported nature of data collected in interviews with respondents. Potential sources of bias may arise from self-reported data; these include attribution, whereby respondents may attribute positive events and outcomes to their own agency, but also the attribution of negative events and outcomes to external forces eg, lack of promotion. However, the sentiments expressed by the COs are congruent with other studies; therefore, attribution bias can be assumed to be minimal. There could also be a bias resulting from participants knowing that the researchers were part of the team that implemented the COST-Africa project. However, this study did not focus on any aspect of evaluation of the project; and the interviewer was not a member of and not involved in implementation of either project; therefore such bias is likely to be minimal. Finally, this study did not employ triangulation of data collection methods, using interviews as the only method to capture data. Interviews were used in order to capture a wide range of perspectives, opinions and ideas and were the most appropriate method to inform the research question.

## Acknowledgements

 The SURG-Africa project is funded by the European Union’s Horizon 2020 Programme for Research and Innovation, under grant agreement no: 733391.

## Ethical issues

 The study was reviewed and approved by the University of Malawi College of Medicine Research Ethics Committee (ref no.: P.03/12/1188) and amended to further include the scope of this study (ref no.:P.01/18/2336). This study was also reviewed and approved by the Research Ethics Committee of the Royal College of Surgeons in Ireland (ref no.: REC727) and Maastricht University (ref no.: FHML/GH_2019.039).

## Competing interests

 Authors declare that they have no competing interests.

## Authors’ contributions

 JG helped conceive the original idea and study design; helped with data acquisition, analysis, and interpretation; and helped review the literature, write the first draft of the manuscript, and approve the final manuscript. MW helped with data acquisition, analysis, and interpretation; and helped review the literature, write the first draft of the manuscript, and approve the final manuscript. CP helped conceive the original idea and study design; helped with data acquisition, analysis, and interpretation, and critically appraised and approved the final manuscript. GM helped with data acquisition, and critically appraised and approved the final manuscript. EB, LB, and RB helped conceive the original idea and study design, and critically appraised and approved the final manuscript.

## Authors’ affiliations

^1^Institute of Global Surgery, Royal College of Surgeons in Ireland, Dublin 2, Ireland. ^2^Faculty of Health, Medicine and Life Science, Maastricht University, Maastricht, The Netherlands. ^3^Division of Population Health Sciences, Royal College of Surgeons in Ireland, Dublin 2, Ireland. ^4^Department of Surgery, College of Medicine Malawi, Blantyre, Malawi. ^5^Radboud University Medical Centre, Nijmegen, The Netherlands.
